# Eletromagnetic Detection of Mild Brain Injury: A Novel Imaging Approach to Post Concussive Syndrome

**DOI:** 10.4236/jbise.2021.1411030

**Published:** 2021-11-24

**Authors:** James Rizkalla, David Botros, Nasser Alqahtani, Mounica Patnala, Paul Salama, Felipe Pablo Perez, Maher Rizkalla

**Affiliations:** 1 Baylor University Medical Center, Dallas, Texas, USA;; 2 John Hopkins Medicine, Department of Neurology, Baltimore, MD, USA;; 3 Department of Electrical and Computer Engineering, Indiana University Purdue University Indianapolis (IUPUI), Indianapolis, IN, USA;; 4 Department of Medicine, Indiana University School of Medicine, Indianapolis, IN, USA

**Keywords:** Brain Injury, Trauma, Imaging, Simulation, COMSOL

## Abstract

**Introduction:**

Mild traumatic brain injury (mTBI) is a common injury, with nearly 3 – 4 million cases annually in the United States alone. Neuroimaging in patients with mTBI provides little benefit, and is usually not indicated as the diagnosis is primarily clinical. It is theorized that microvascular trauma to the brain may be present in mTBI, that may not be captured by routine MRI and CT scans. Electromagnetic (EM) waves may provide a more sensitive medical imaging modality to provide objective data in the diagnosis of mTBI.

**Methods:**

COMSOL simulation software was utilized to mimic the anatomy of the human skull including skin, cranium, cerebrospinal fluid (CSF), gray-matter tissue of the brain, and microvasculature within the neural tissue. The effects of penetrating EM waves were simulated using the finite element analysis software and results were generated to identify feasibility and efficacy. Frequency ranges from 7 GHz to 15 GHz were considered, with 0.6 and 1 W power applied.

**Results:**

Variations between the differing frequency levels generated different energy levels within the neural tissue—particularly when comparing normal microvasculature versus hemorrhage from microvasculature. This difference within the neural tissue was subsequently identified, via simulation, serving as a potential imaging modality for future work.

**Conclusion:**

The use of electromagnetic imaging of the brain after concussive events may play a role in future mTBI diagnosis. Utilizing the proper depth frequency and wavelength, neural tissue and microvascular trauma may be identified utilizing finite element analysis.

## 1. INTRODUCTION

Sudden head trauma resulting in mild traumatic brain injury (mTBI) has been recognized as a potential injury for centuries [1]. Though there is a long history of recognizing potential damage to the brain with repeated head trauma, only recently in the last 1 – 2 decades, has there been a resurgence in protecting patient’s from repeat head trauma, or injury immediately during the post concussive state [1]. Concussions, or mTBIs, are a significant public health problem and have fostered much attention in recent years from the public, media outlets, and physicians or healthcare providers [2]. Concussions commonly present with headache, fatigue, memory loss, and confusion [3]. However, many of these injuries may be more subtle and may not manifest or present itself until a later time—subsequently putting them at risk for repeat head trauma [3]. Concussions continue be remarkably common and frequent source of morbidity for adolescents and athletes [1, 2, 4]. It is estimated that about 1.6 to 3.8 million concussions occur each year in the United States [1, 3, 4]. Though this remains a common issue for athletes or patients after mild head trauma, the long term effects of concussions remain unknown, though attempt to recognize and diagnose this injury continues to be a goal of physicians and around the world.

From an imaging standpoint, a diagnosis of mTBI continues to be challenging to make using standard imaging techniques, Computed Tomography (CT) and Magnetic Resonance Imaging (MRI). In fact, one of the diagnostic criteria for mTBI is the lack of radiographic abnormalities on neuroimaging [4, 5]. In contrast, major TBIs commonly result in hemorrhage from veins or arteries within the cranium and are readily visualized on CT scan, routinely without contrast. MRI imaging after a major head trauma can demonstrate hemorrhage, perfusion defects, or even diffuse axonal injury. However, routine CT scans and MRIs are ineffective in the diagnosis of mild traumatic brain injuries. Therefore, assessment tools such as the Sport Concussion Assessment Tool (SCAT), ImPACT, and numerous others have been developed to assist in developing the clinical diagnosis of post-concussive syndrome [2, 4, 6].

Though assessment tools, as well as clinical examination, continue to be the mainstay in diagnosing mTBI, newer technologies are attempting to image patients in the post-concussive state to provide objective data for adequate diagnosis. Some promising technologies have been developed, such as, diffusion tensor imaging, positron emission tomography, perfusion imaging, and H-magnetic resonance spectroscopy [2]. Experimental advanced MRI scans have demonstrated some efficacy in identifying damage to neurologic microvasculature—otherwise not captured by standard CT, MRI, or other neuroimaging [2]. Though promising, the cost, portability, and accessibility of these new imaging processes remain unknown and are likely to present obstacles in widespread use. For example, the United States’ healthcare system increasing focus on value-based medicine may render advanced MRIs, perfusion imaging, and PET scans inappropriate. Additionally, recognizing that these injuries commonly occur in adolescents, minimizing radiation is a priority. Therefore, there is an opportunity to investigate an imaging modality to identify microvasculature lesions after minor head trauma in a cost-efficient, radiation-free, and accessible fashion. This study utilizes a simulation, as a feasibility model, to investigate the use of electromagnetic radiation as a novel imaging approach to recognizing microvascular trauma [7] in the post-concussive state [8].

## 2. METHODS

**Figure 1.**
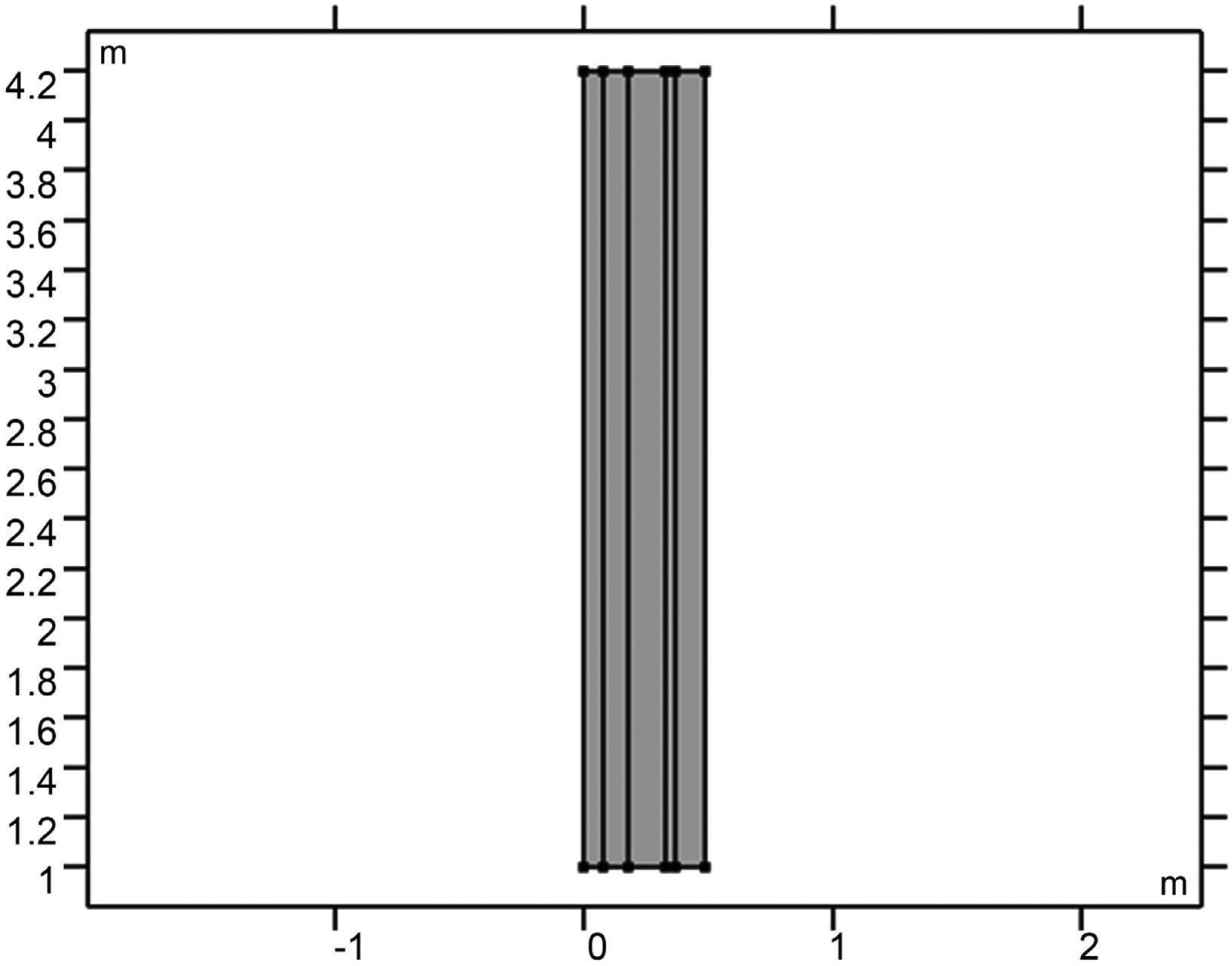
2D model for five layers considered for the study. Skin tissue is from the left with the brain tissue at the far right.

**Figure 2.**
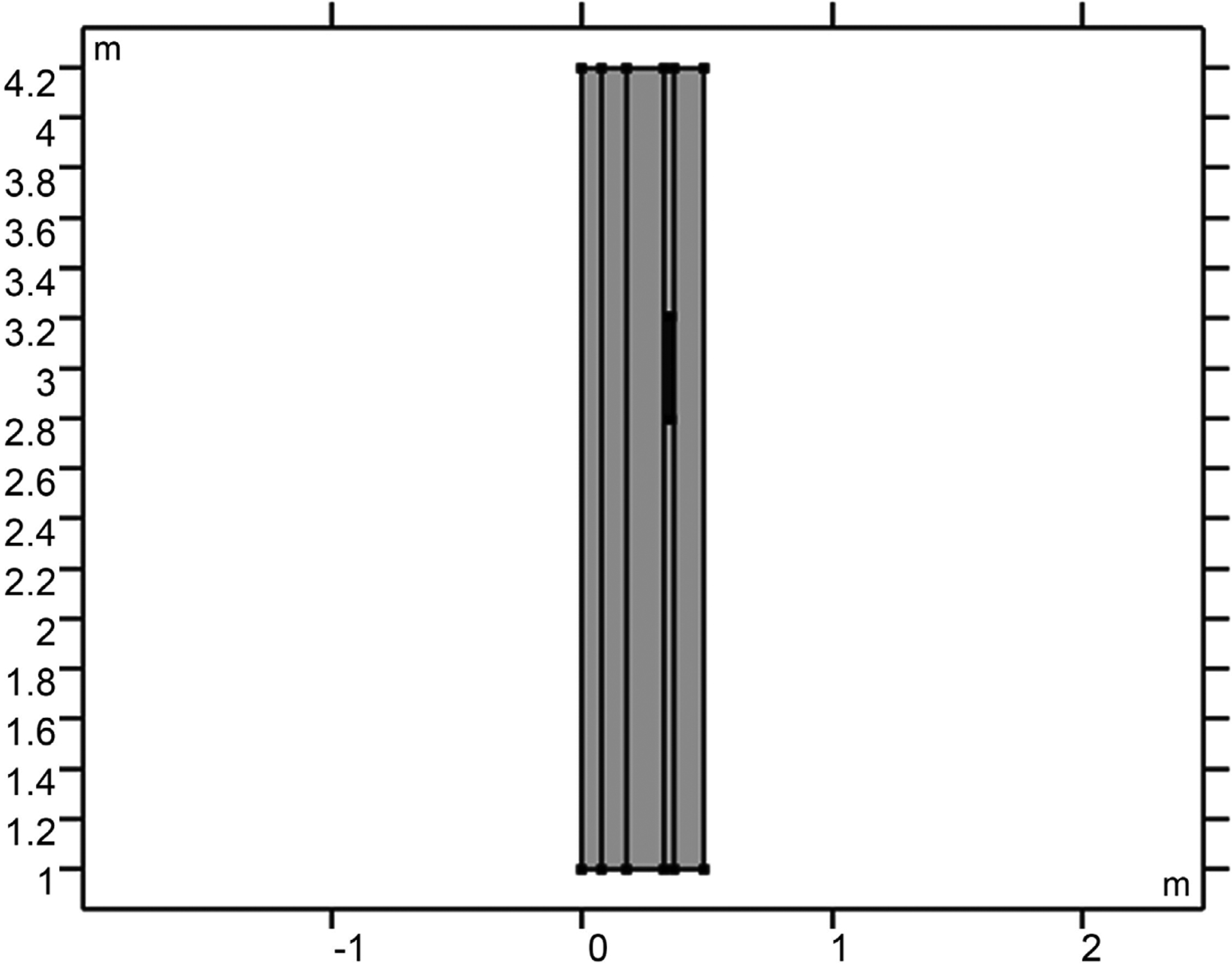
2D model for five layers considered for the study with raptured artery.

**Figure 3.**
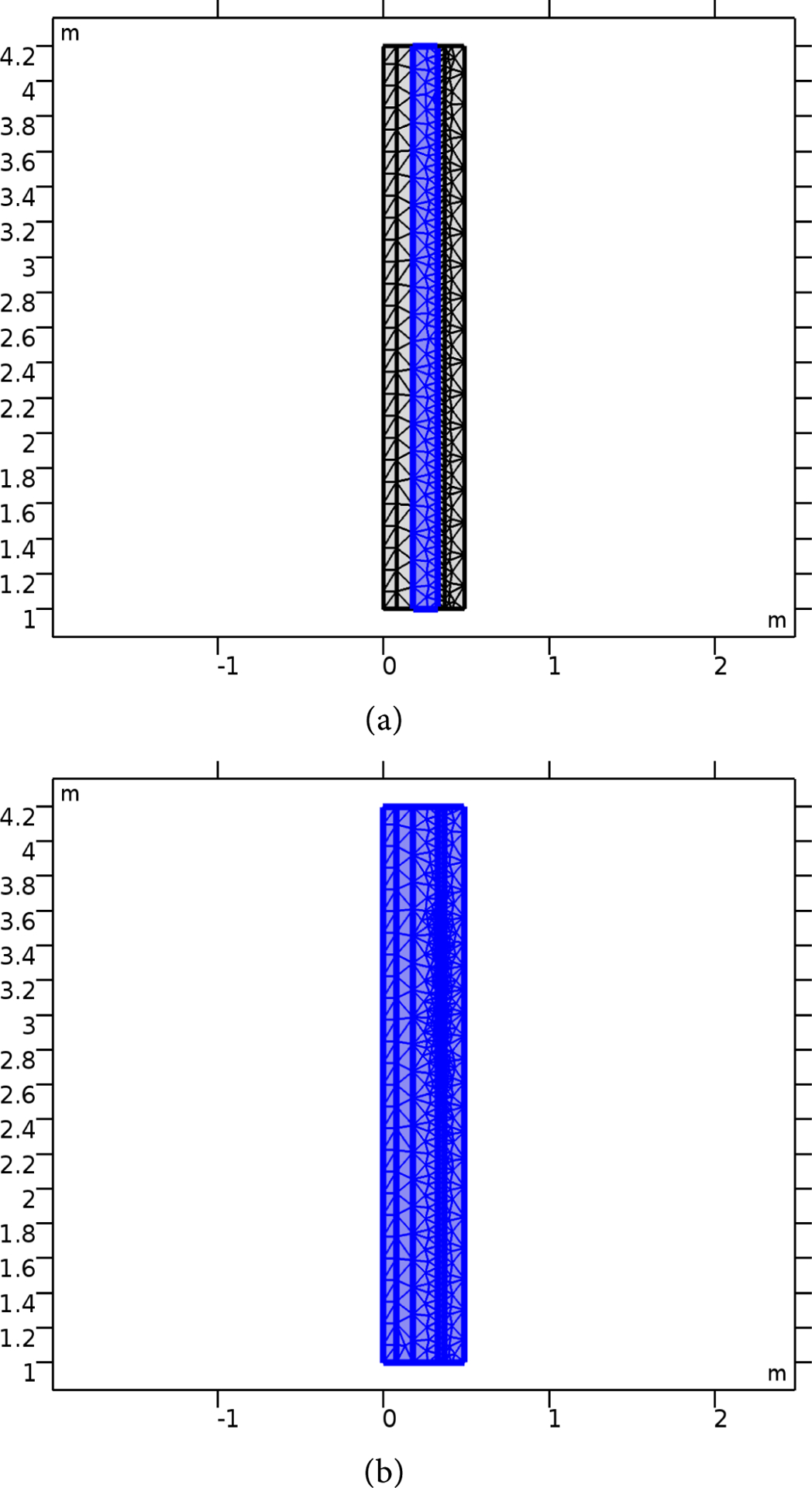
The Mesh model used for the study. (a) The mesh layers with artery rapture; (b) shows the case with no rapture, the blue color covers the cerebrospinal fluid CSF layer followed by the artery layer from the right.

COMSOL Multiphysics software was utilized in the investigation of electromagnetic application on human tissues. A model was developed to simulate the impact of the flow of electromagnetic waves on a model containing layers which mimic the human skull: the skin, skull, cerebrospinal fluid (CSF), brain, and microvasculature. Figure 1 shows the 2D simulation model used in the study. The 2D model with rapture artery is shown in Figure 2. The size used here is in the cm range, making it suitable for a practical model. The COMSOL mesh models for both raptured and normal are given in Figure 3.

## 3. EM MODELING

The wave equation and the parameters associated with the simulation model are given as follows:

(1) $$\nabla \times \mu_{r}^{-1}(\nabla \times E)-k_{0}^{2}\left(\epsilon_{r}-\frac{j\sigma }{\omega \epsilon_{0}}\right)E=0$$

where *σ* is the conductivity of the material, *ω* is the radian frequency; *ϵ_r_* is the relative permittivity of the material. The boundary condition at the sample was obtained by matching the tangential components of the electric fields using *n* × *E* = 0 at the interfaces. Impedance Boundary Condition equations are given by:

(2) $$\sqrt{\frac{\mu_{0}\mu_{r}}{\varepsilon_{0}\varepsilon_{r}-\frac{j\sigma }{\omega }}}n\times H+E-(n\cdot E)n=\left(n\cdot E_{s}\right)n-E_{s}$$

The equations governing the magnetic field study are:

(3) ∇×H=J

(4) B=∇×A

(5) J=σE+jωD+σ(v×B)+Je

(6) E=−jωA

## 4. RESULTS AND DISCUSSIONS

Vasculature that was studied in this simulation was designed to mimic the human brain’s microvasculature. Micro-hemorrhage was simulated using the appropriate vessel diameter, circumference, and overall features. Various frequencies were considered in order to find the best fit in penetration depth via the multilayers given above. The ranges of frequencies from 7 GHz to 15 GHz were simulated in order to penetrate and detect damage to the microvasculature. Table 1 shows the characteristics of the material used in the simulation. The study of the status of the vessel inside the human brain was addressed via the solution of the wave equation within the multiple layer structure. Wavelength *λ*, depth of penetration *δ*, power in watt, and widths of various layers (skin, skull, CSF, blood vessels, grey matter of brain) were important parameters in this study. The transmission characteristics of the materials have resulted in the differential analysis of the vessels within the brain parenchyma. Three frequencies were considered: 7 GHz, 10 GHz, 15 GHz. With the material transmission properties within the COMSOL software, the frequencies used in this investigation were suitable. The energy levels considered were 0.6 W, 1 W and 10 W. The low-energy pulses were appropriate for researching the transmission features and layer interface within the 2D structure. Different simulation results were obtained in response to the penetration depths of the tissue materials.

**Figure 4.**
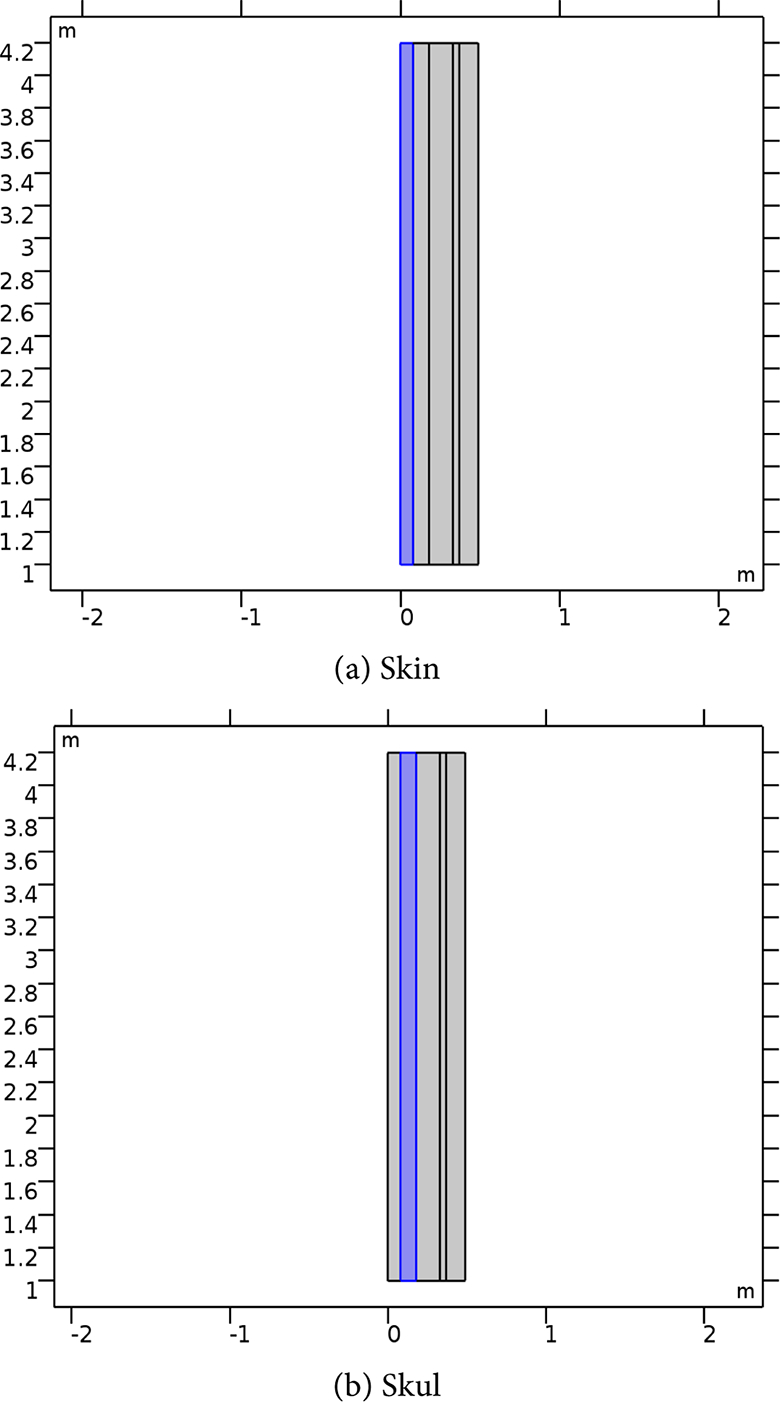

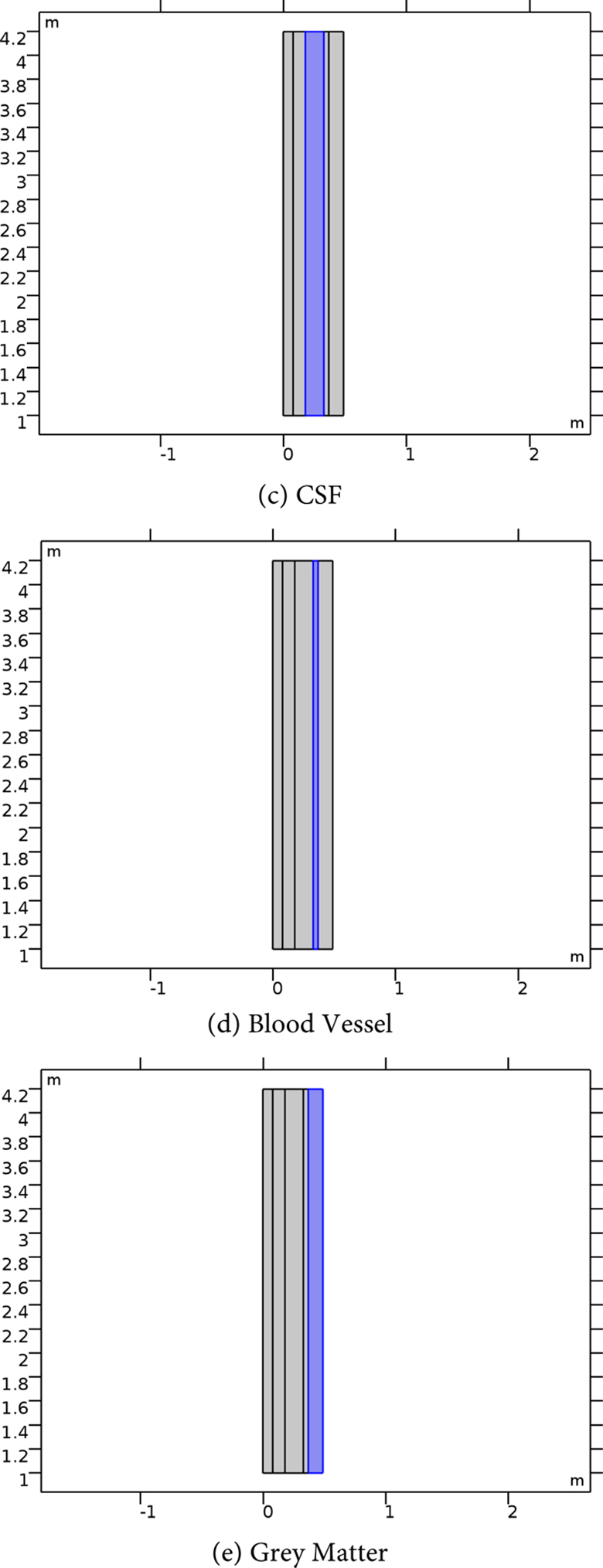
The various layers of the multiple structure. CSF is cerebrospinal fluid.

**Figure 5.**
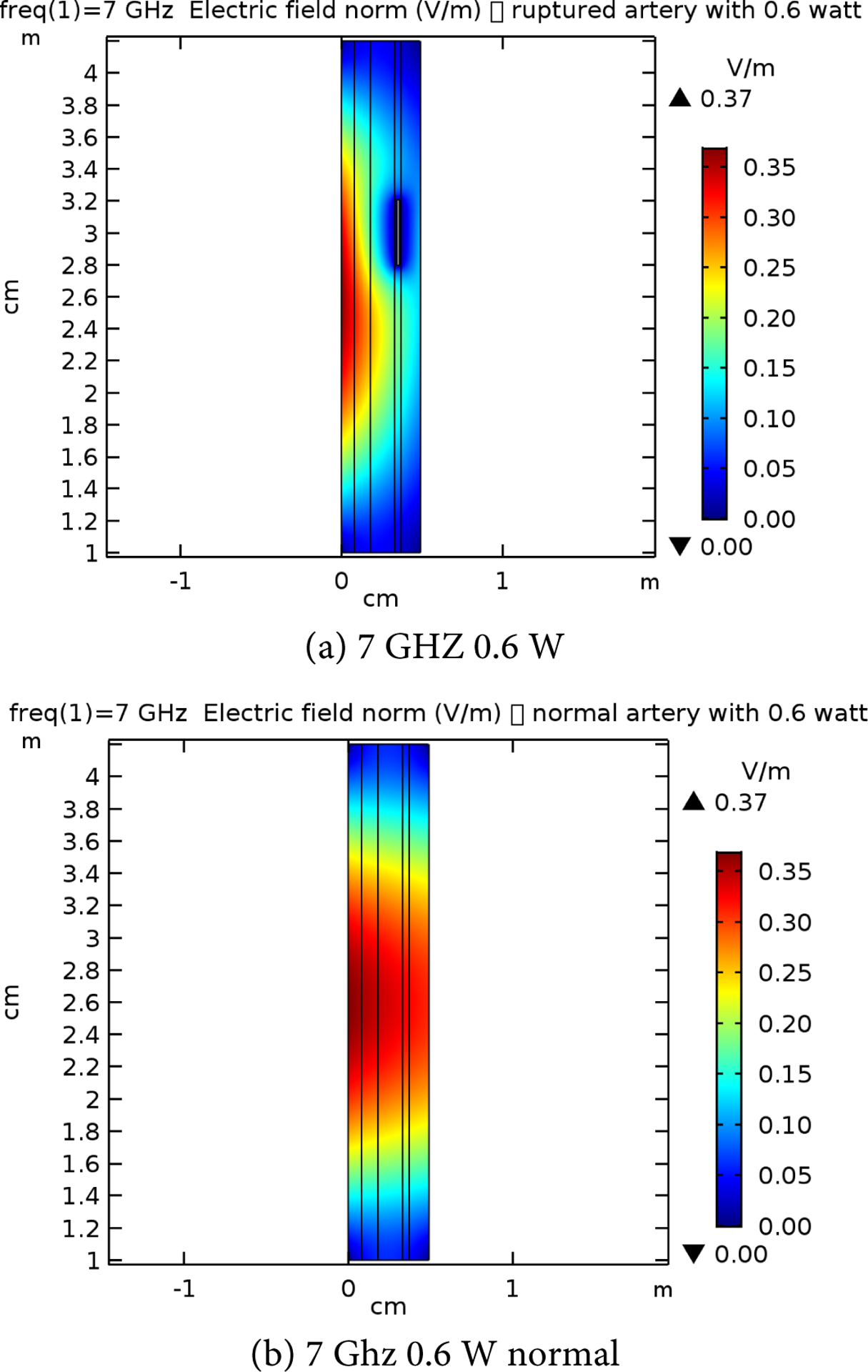
E-field simulation results for 7 GHz at 0.6 W power for (a) normal and (b) raptured tissues.

**Figure 6.**
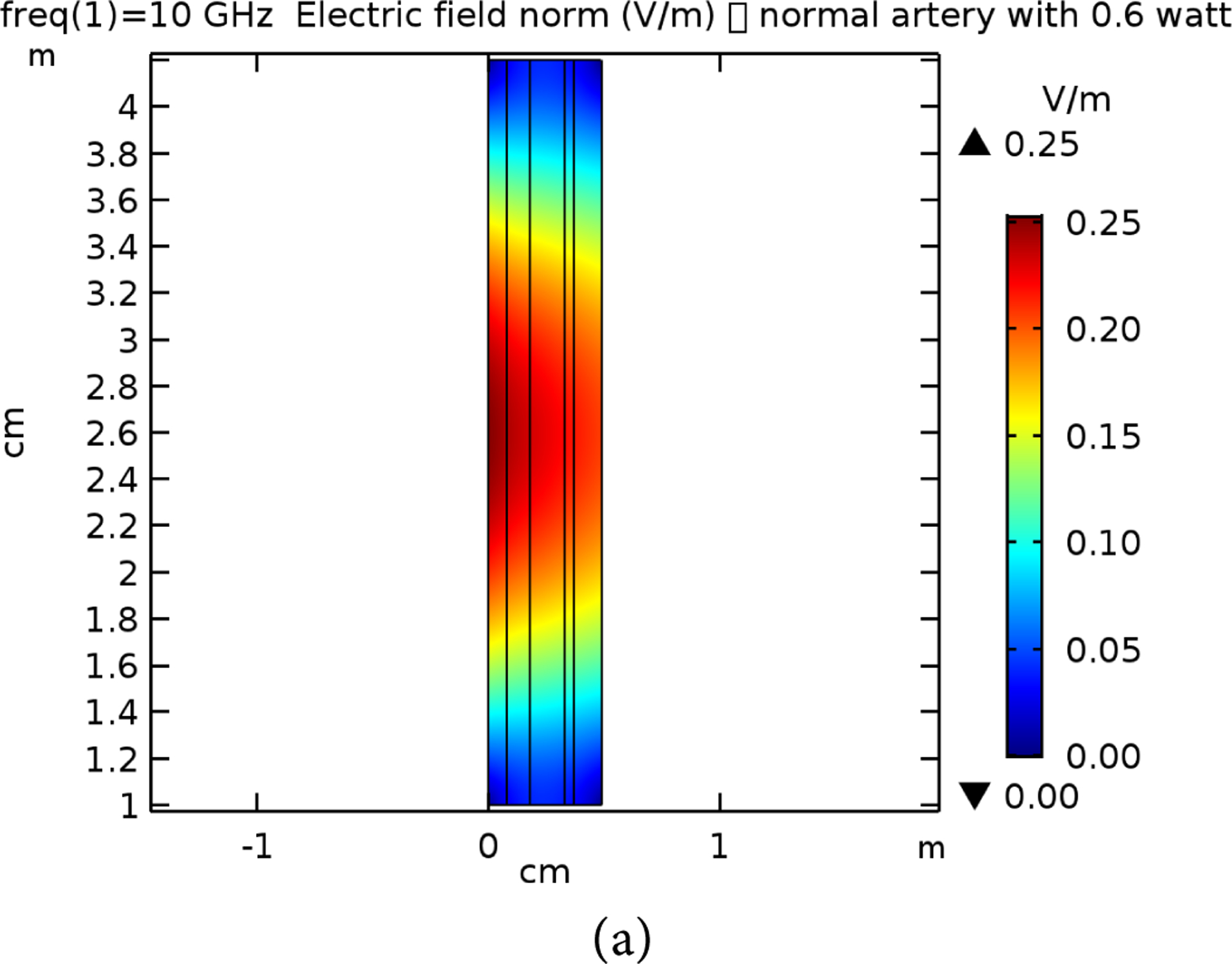

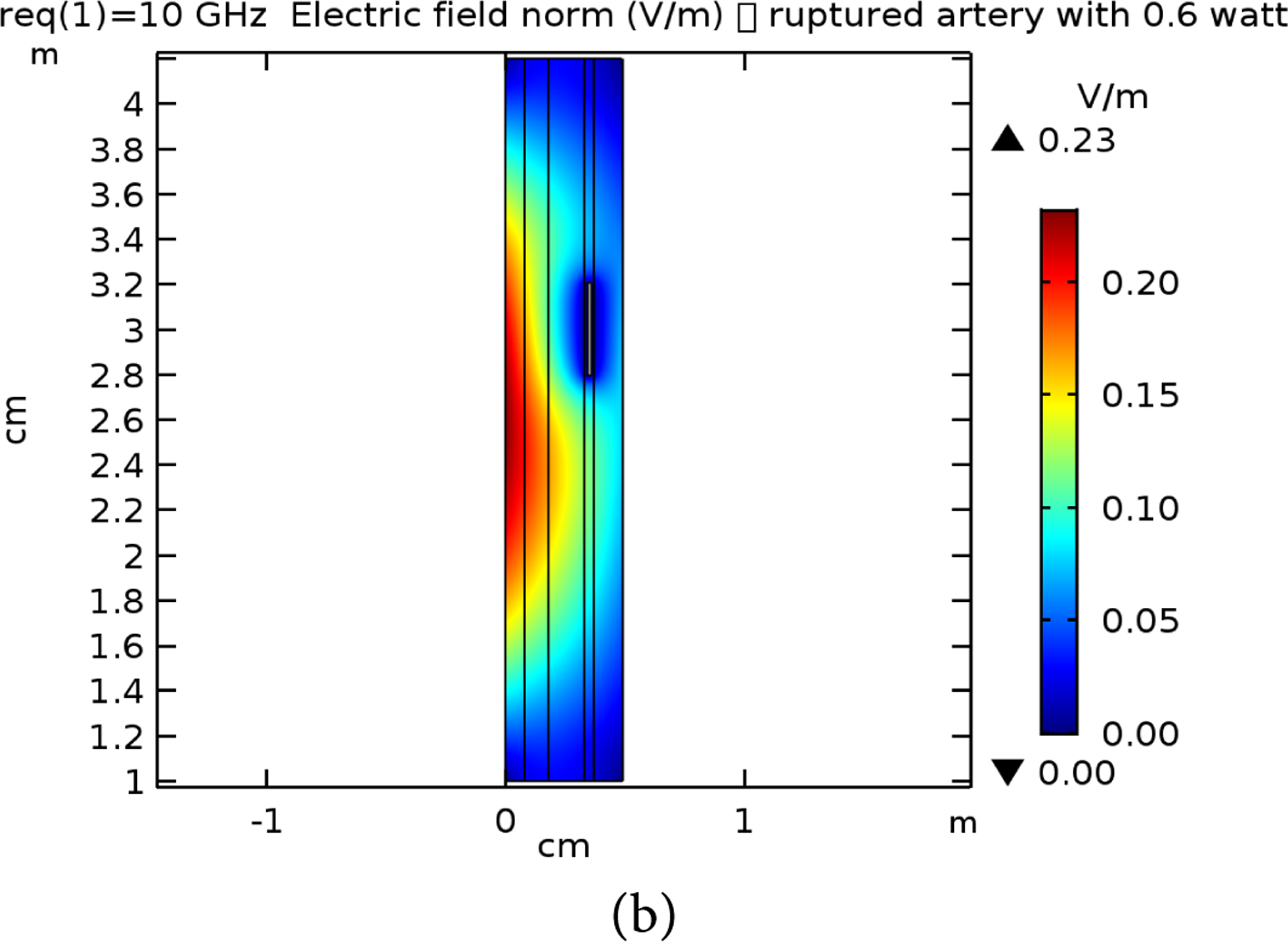
E-field simulation results for 10 GHz at 0.6 W power for (a) normal and (b) raptured tissues.

**Figure 7.**
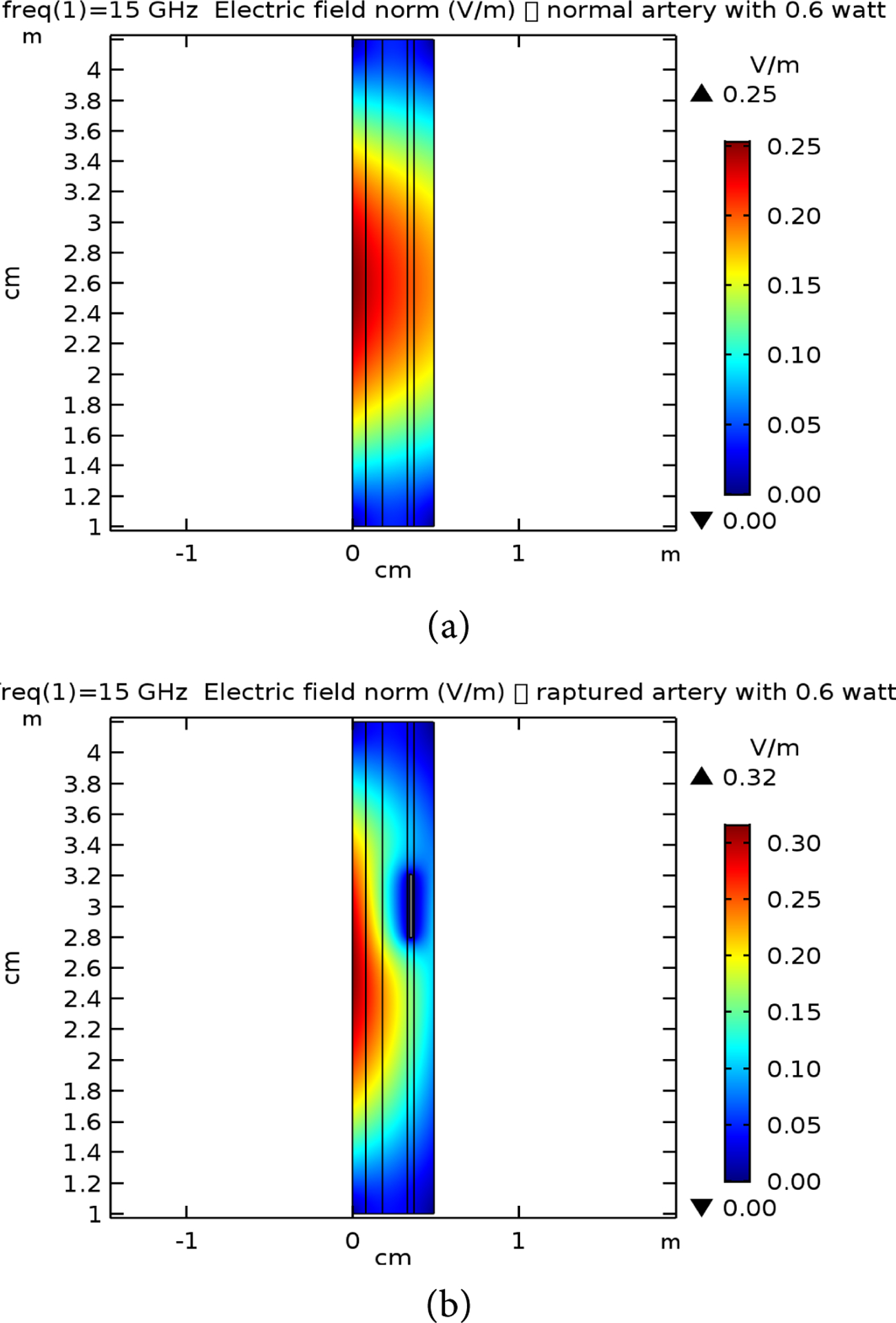
E-field simulation results for 15 GHz at 0.6 W power for (a) normal and (b) raptured tissues.

**Figure 8.**
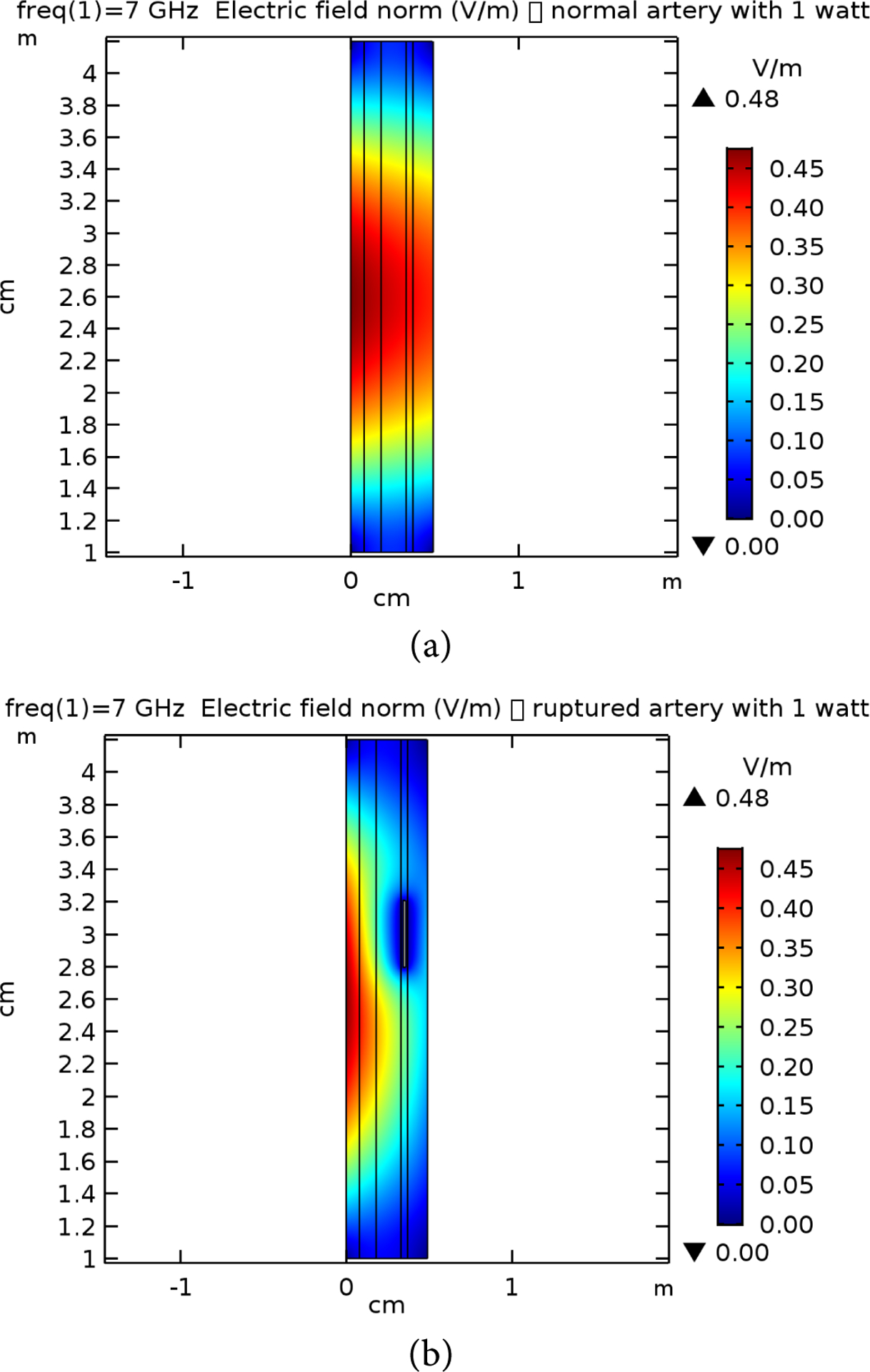
E-field simulation results for 7 GHz at 1 W power for (a) normal and (b) raptured tissues.

**Figure 9.**
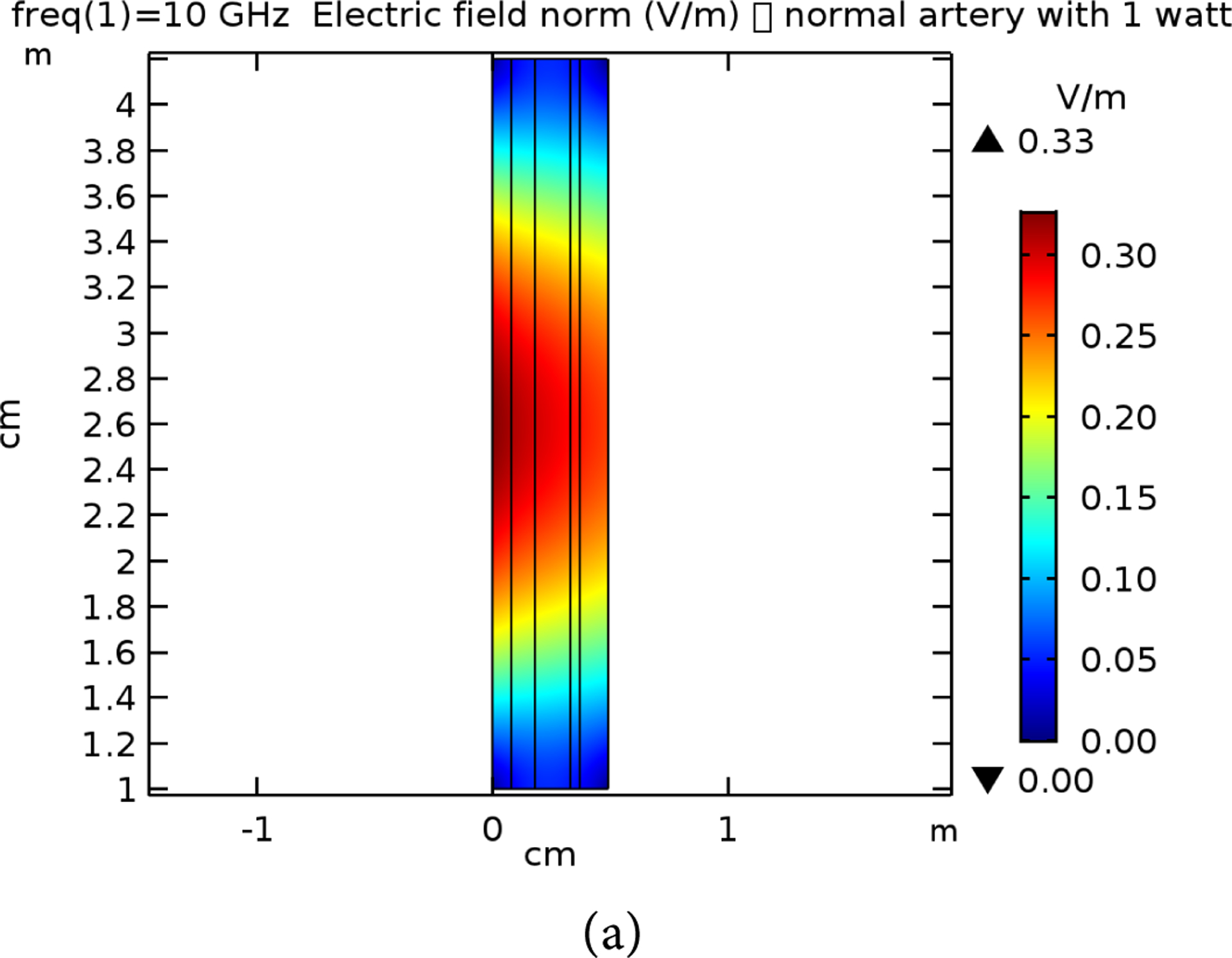

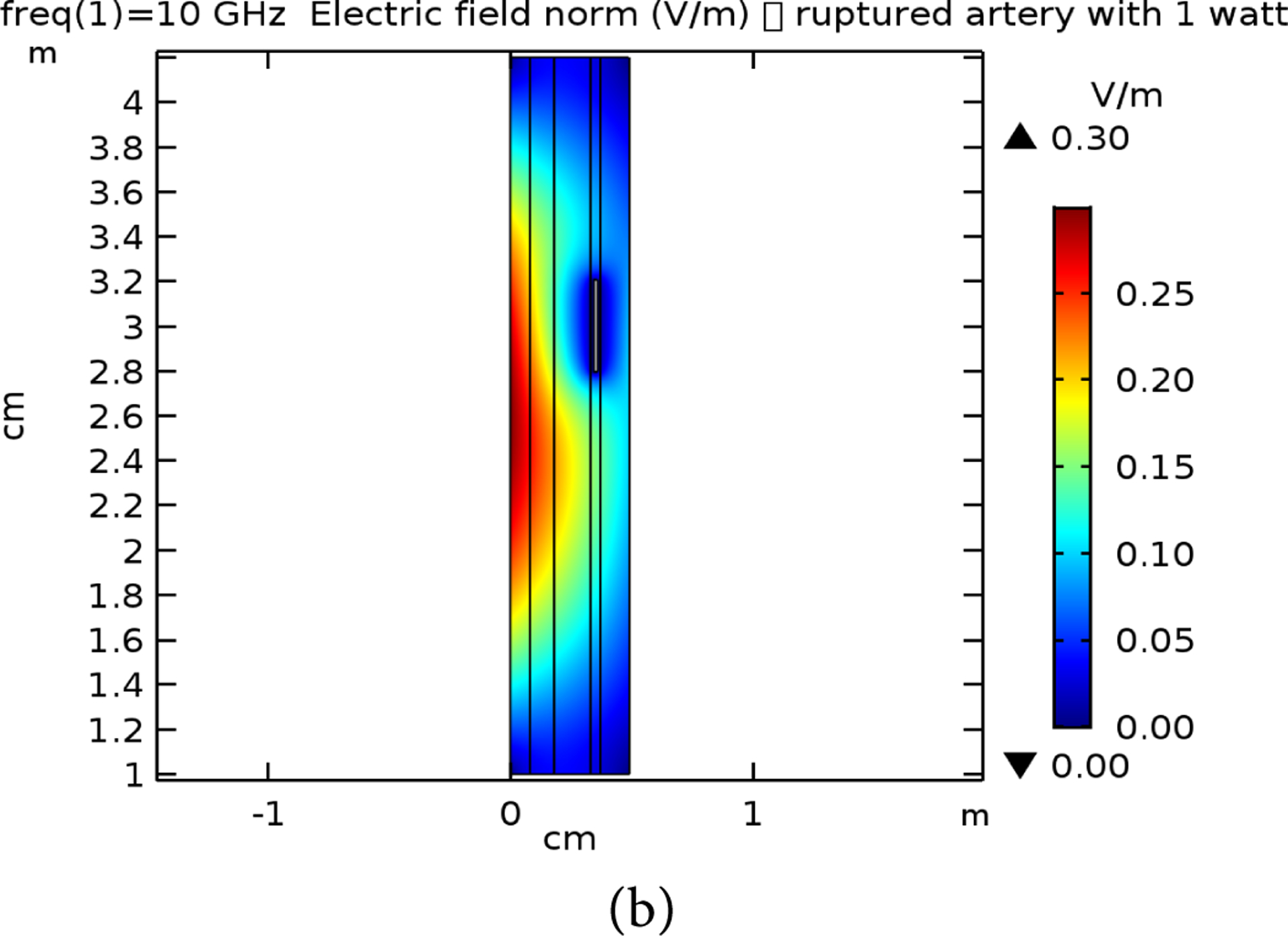
E-field simulation results for 10 GHz at 1 W power for (a) normal and (b) raptured tissues.

**Figure 10.**
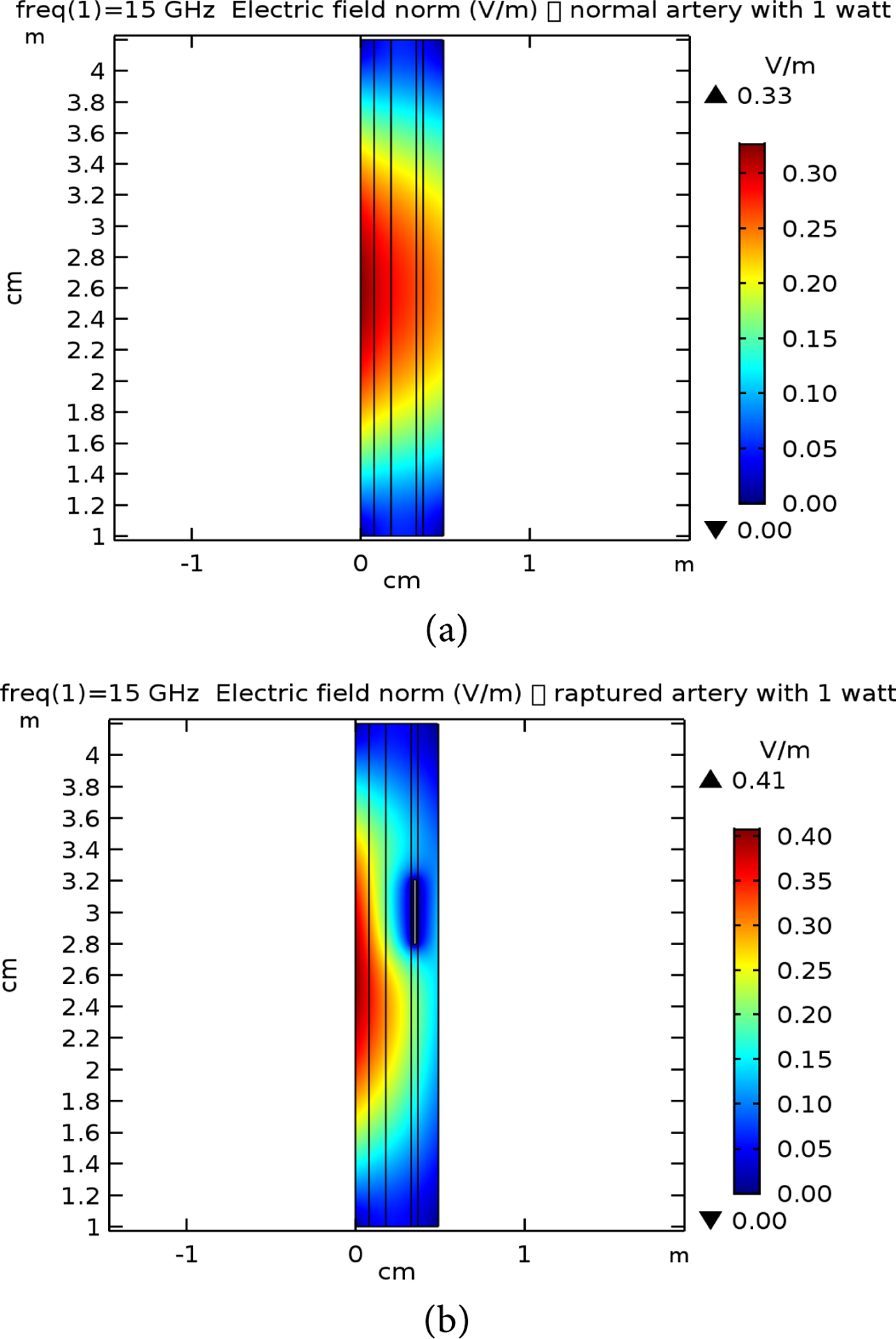
E-field simulation results for 15 GHz at 1 W power for (a) normal and (b) raptured tissues.

**Figure 11.**
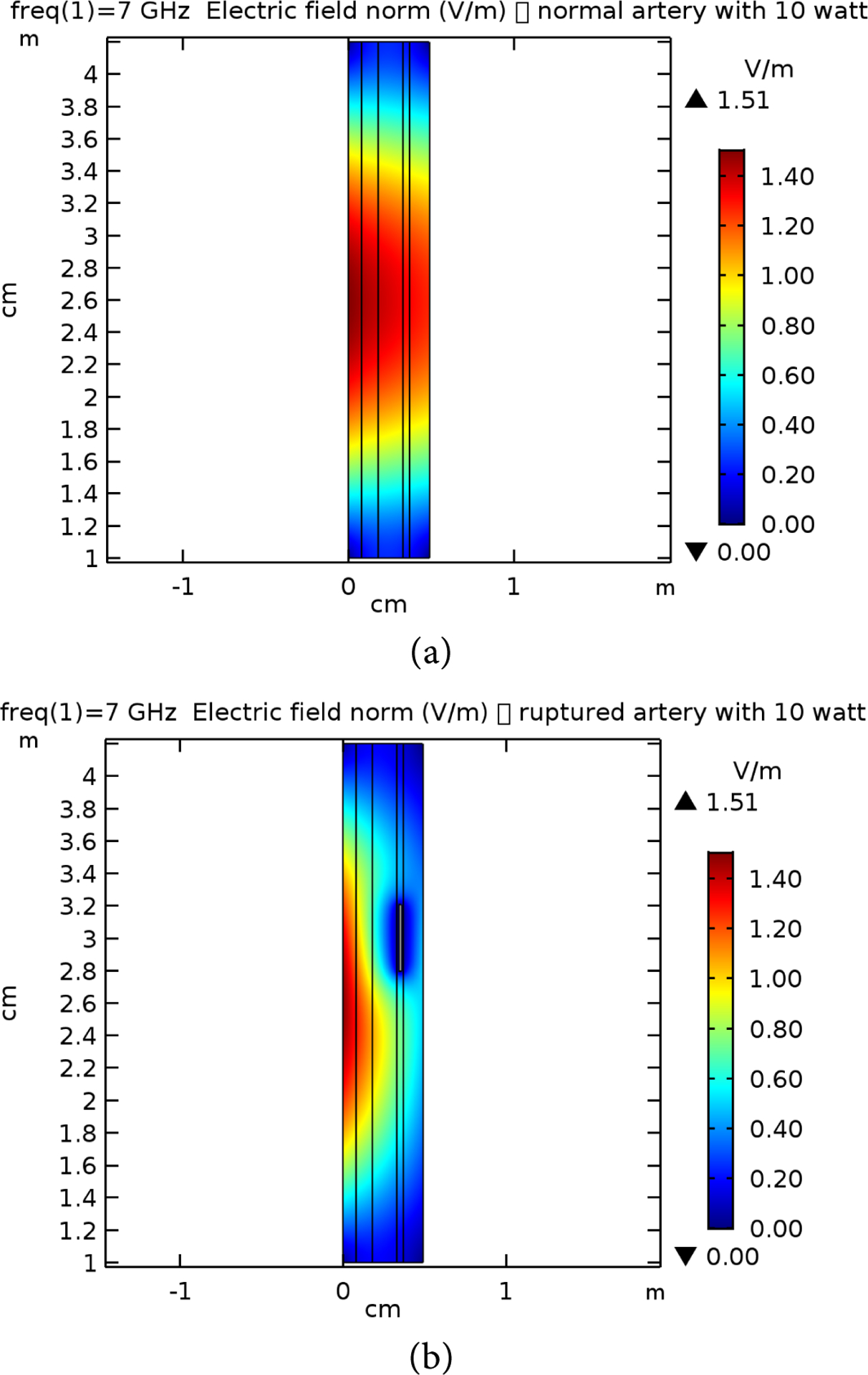
E-field simulation results for 7 GHz at 10 W power for (a) normal and (b) raptured tissues.

**Figure 12.**
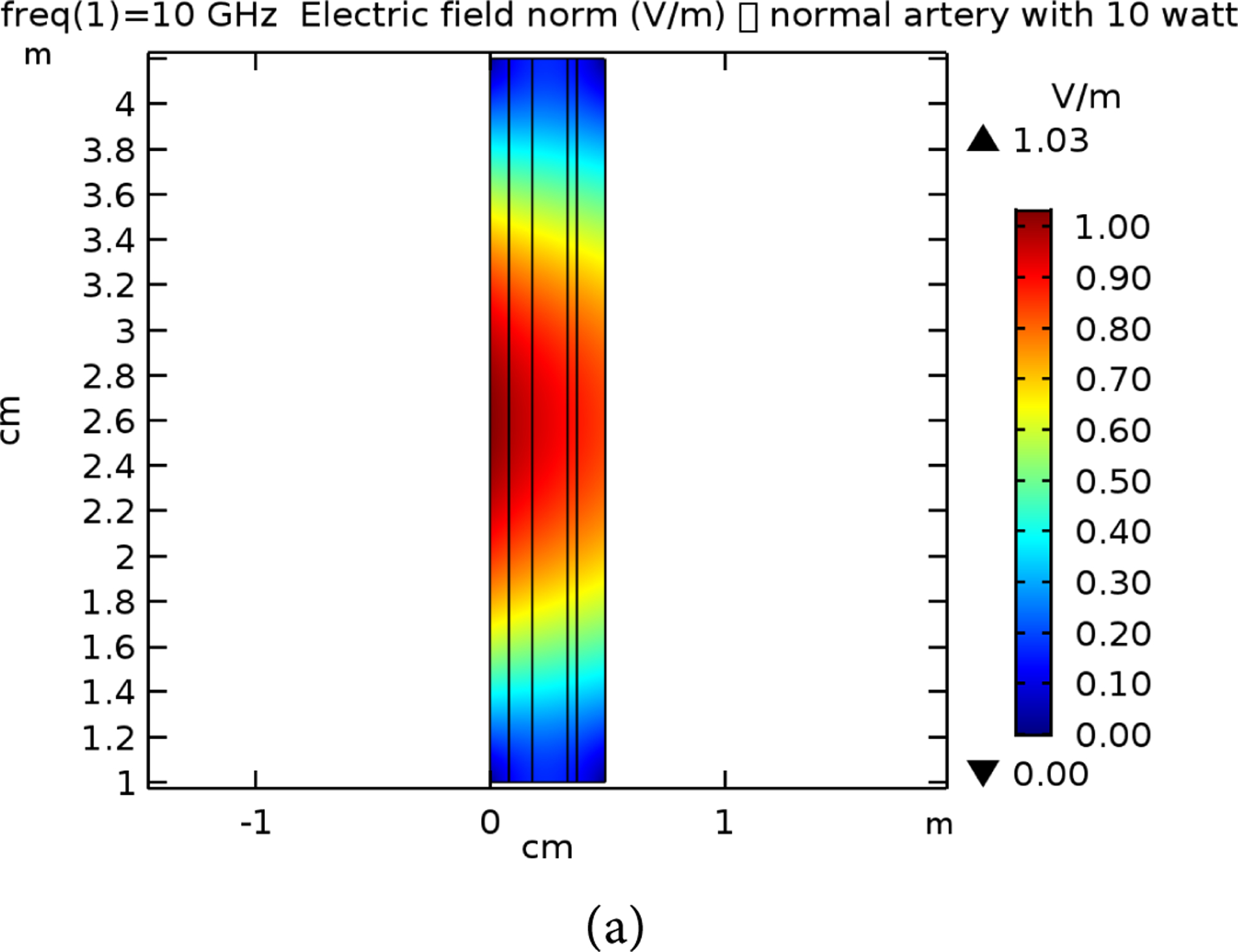

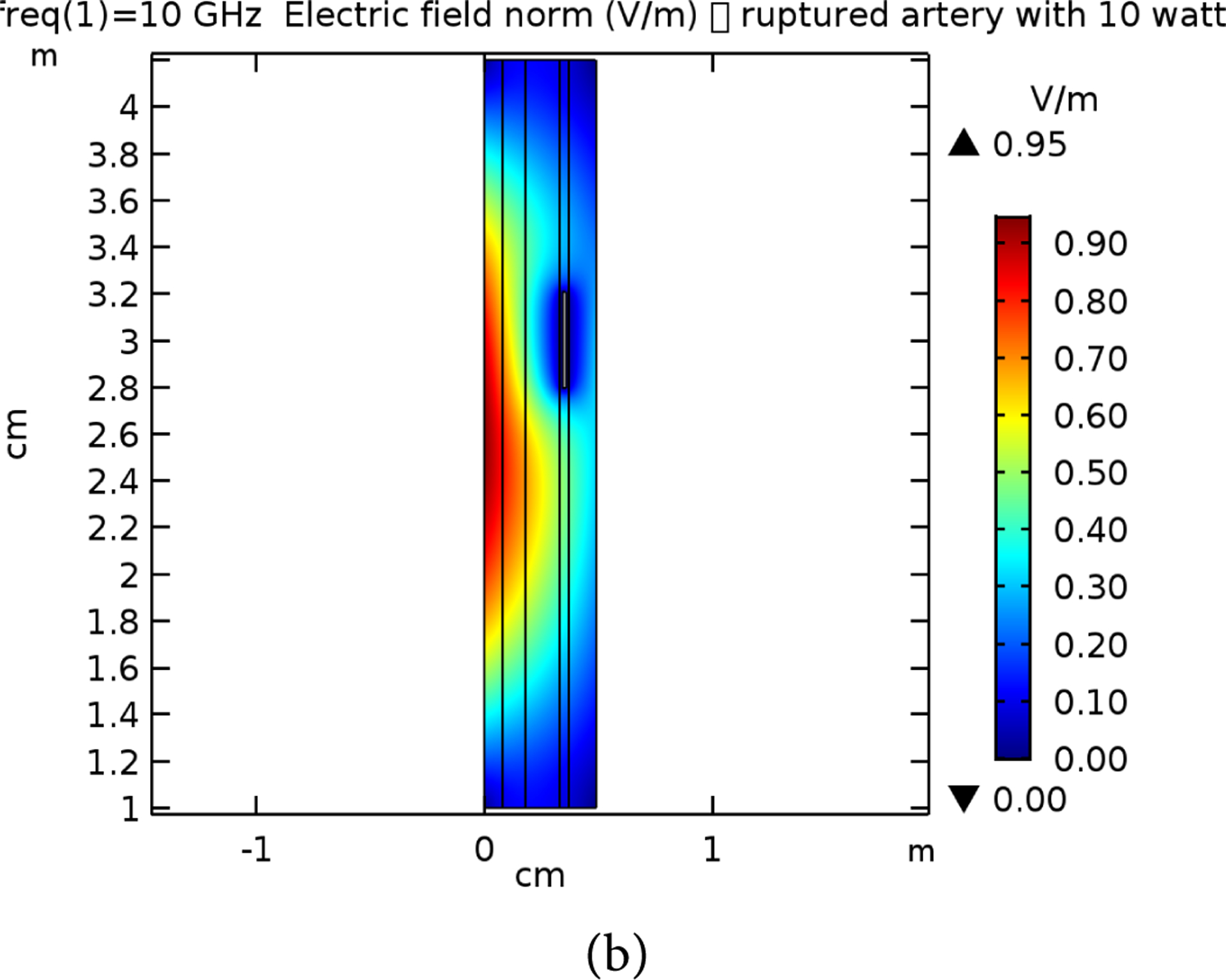
E-field simulation results for 10 GHz at 10 W power for (a) normal and (b) raptured tissues.

Table 2 presents the different layer sizes of the multiple structure used in the study, and all presented in Figure 4. These are used for references as compared to the impact of the frequency and power applications. The E-simulation for 0.6 W for 7 GHz, 10 GHz, and 15 GHz for both normal and raptured tissues are given in Figures 5–7. The impact of 1 W power for both normal and raptured tissues for the three frequencies, 1 GHz, 7 GHz, and 10 GHz are given in Figures 8–10. Figure 11 and Figure 12 present the impact of 10 W power for the same frequency range.

## 5. CONCLUSION AND FUTURE WORK

**Figure 13.**
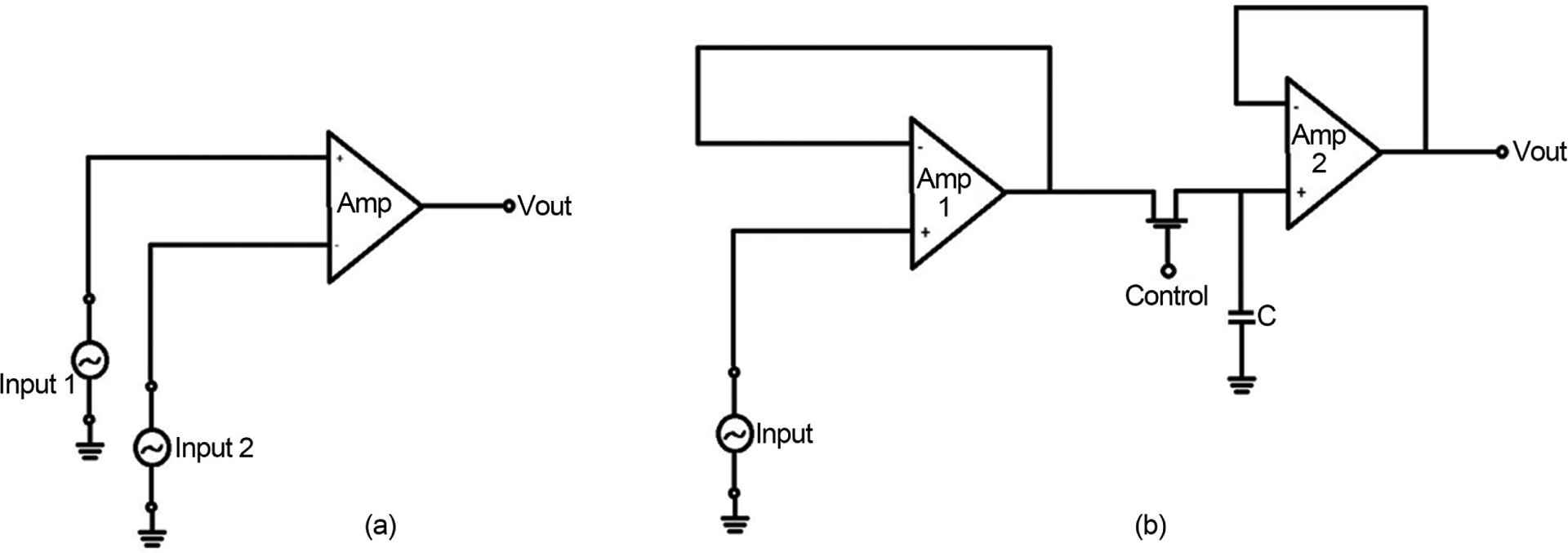
The proposed electronic circuits for the practical model representing. The left circuit is a comparator circuit for generating the digitized output. The right circuit is the sample and hold off circuit to store the data within the capacitance.

In this present work, we demonstrated the concept of detecting microvasculature in the brain using EM waves and its potential in diagnosing mTBI. Given the challenge in diagnosing concussions without an imaging modality, EM provides an opportunity to do so without the expense of MRI or the radiation involved with CT scans. The figures obtained from the transmitted and reflected areas as determined by COMSOL’s finite element simulation are within the MEMS (micro-electro-mechanical systems) current sensor technology. For better measurements, the Hall Effect sensors can be constructed on flexible substrates to shape up the sensors around the head. Figure 13(a) shows a comparator circuit that may be used to provide a digitized formatted output. Input 1 could be a reference potential, while input 2 could be the signal reflecting the information with the traumatic brain injury. The output of the comparator will be the signal fed into the sample and hold off circuit, representing the signal coming from the sensors. Buffering circuitries may be necessary for better interfacing the sensor to the circuit as shown in Figure 13(b).

The EM approach proposed in this work is novel in the sense that a phase array (PA) antenna may be utilized to control the directivity of major beam towards the brain tissue. The sharp edge of the beam enables the investigation of a target area in human tissue. The small size of the antenna at the GHz range will be appropriate for integrating it with the magnetic sensors, forming a system on chip (SOC) for such application. The study of integrating EM seniors with high directivity PA antenna is reserved for future consideration. Though this is a promising start towards a future method of imaging, this remains a largely unexplored modality. Future studies transitioning from a realistic human head voxel phantom [9] simulation to a practical model with a specific anthropomorphic mannequin [10] are a logical next step in progressing this technology towards ultimate utilization within patient care.

## Figures and Tables

**Table 1.** Material properties used in the simulation geometry statistics.

Material	Frequency	Relative Permittivity	Elec’ Conductivity
Skin			
	7 GHz	3.41 E+1	4.82 E+0
	10 GHz	3.31 E+1	8.01 E+0
	15 GHz	2.64 E+1	1.38 E+1
Skull			
	7 GHz	9.17 E+0	1.44 E+0
	10 GHz	8.12 E+0	2.14 E+0
	15 GHz	6.87 E+0	3.15 E+0
CSF			
	7 GHz	5.85 E+1	9.86 E+0
	10 GHz	5.24 E+1	1.54 E+1
	15 GHz	4.37 E+1	2.47 E+1
Artery			
	7 GHz	3.66 E+1	5.65 E+0
	10 GHz	3.27 E+1	9.13 E+0
	15 GHz	2.64 E+1	1.48 E+1
Grey Matter			
	7 GHz	4.23 E+1	6.42 E+0
	10 GHz	3.81 E+1	1.03 E+1
	15 GHz	3.19 E+1	1.69 E+1

**Table 2.** Width of layers used in the simulation.

	Skin	Skull	CSF	Blood Vessel	Grey Matter
**Width (cm)**	0.08	0.1	0.15	0.04	0.12
**Height (cm)**	3.2	3.2	3.2	3.2	3.2
